# Health equity and health system strengthening – Time for a WHO re-think

**DOI:** 10.1080/17441692.2020.1867881

**Published:** 2021-01-10

**Authors:** N. Jensen, A. H. Kelly, M. Avendano

**Affiliations:** a Department of Global Health and Social Medicine, King’s College London, North East Wing, 40 Aldwych, London, WC2B 4BG, UK; b Department of Social and Behavioral Sciences, Harvard T.H. Chan School of Public Health, Boston, USA

**Keywords:** Equity, health systems, World Health Organization, social determinants, social justice

## Abstract

The pursuit of health equity is foundational to the global health enterprise. But while moral concerns over health inequities can galvanise political commitment, how such concerns can or should translate into practice remains less clear. This paper reviews evolving ways that equity goals have featured in key World Health Organization (WHO)-related policy documents, before discussing the heuristic value and empirical traction that the concept of equity can bring to the health system strengthening (HSS) agenda. We argue that while health equity is often presented as the overarching goal of HSS, in practice this is typically circumscribed to the provision of healthcare services. Although health*care* equity is important, we suggest that this narrow focus risks losing sight of the structural political, social and economic drivers of health and health inequities, as well as the broader contexts of care and complex socio-political mechanisms through which health systems are strengthened. Drawing on new lines of empirical inquiry, we propose that broadening the equity lens for HSS ­offers exciting opportunities to put health systems at the heart of a more ambitious equity agenda in global health.

## Introduction

In an ever-changing landscape of actors, projects and priorities, the struggle for health equity has served as an enduring and unifying goal of international health efforts. Indeed, in many ways it is what provides the post-Westphalian global health enterprise with its raison d’être: in a multipolar world, the pursuit of collective wellbeing compels international intervention and global collaboration and provides its moral thrust (Koplan et al., 2009). And yet, today the goal of achieving health equity appears as far out of reach as ever (Shamasunder et al., 2020; The Lancet Global Health, 2016). More so, despite 40-odd years of collective statements of purpose on the importance of addressing health inequities, how exactly this goal should be understood and what form the collective response should take remains a matter of heated debate.

One of the most widely adopted definition of health inequities was put forward in 1991 by Margaret Whitehead, who specified that *health equities* refer to health differences that are objectionable from a moral or ethical point of view because they are ‘unnecessary, avoidable, and unfair or unjust’ (Whitehead, 1991, p. 219). Whitehead’s triad remains a prominent short-hand definition of health inequities in the global health literature. And yet, a well-recognised shortcoming is precisely the difficulty in determining when differences in health are indeed unnecessary, avoidable, or unfair: the required judgement depends not only on the specific context in which the judgement takes place, but also on a wider set of normative commitments and ethical principles to arbitrate which health differences are deemed unfair, and why (Braveman et al., 2011; Venkatapuram, 2016). This lack of conceptual clarity is often argued to be a major obstacle that hinders the operationalisation of equity as a guide to health policy and practice, which is why efforts to clarify and harmonise existing equity definitions and interpretations continue (e.g. Braveman et al., 2017; McCartney et al., 2019).

While we acknowledge the political significance and theoretical nuance of this argument, this paper considers the intangibility of health equity from a somewhat different angle. Our argument begins from the proposition that the conceptual elasticity of equity is a key dimension of its practical power. A ‘boundary object’ (Star & Griesemer, 1989) par excellence, equity is abstract enough to capture a number of distinct moral commitments and concern and, thus, provides a normative orientation for global health action undertaken by a variety of actors – e.g. researchers of various disciplines, governments and policymakers, agencies and organisations, non-governmental organisations and civil society groups, health activists, and philanthropists. But the very same latitude that grants equity its pervasive acceptance as aspirational goal also renders it cumbersome and fragile in the field of implementation – it is when it comes to the design of policies, programmes and practices that controversies over the meaning of equity and its realisation arise.

What this paper argues is that efforts to transcend such enduring disagreements and produce a consensus over equity as an actionable and measurable goal have contributed to an effective narrowing of its conceptual scope and programmatic reach. As Amartya Sen has argued, the problems of adhering to a ‘particularly confined interpretation of health equity do not typically lie in the relevance of what that interpretation asserts (this is, often enough, not in doubt), but rather in what it denies’ (Sen, 2002, p. 664). Indeed, what this paper seeks to argue is that efforts to address equity as a measurable goal of health systems strengthening (HSS) have been paralleled by a curtailing of its ideational clout: an increasing focus on health*care* equity has progressively divorced these efforts from a broader social justice agenda that sees health equity as a social, rather than just a biomedical goal.

This paper was motivated by ongoing debates over the prioritisation of Universal Health Coverage (UHC). In recent years, both the strengthening of health systems and the achievement of equity have become increasingly tethered to the over-arching goal of UHC (Astana Declaration, 2018). And yet, many critics point to this prioritisation of UHC as a reflection of the abandonment of a more ambitious social justice agenda that treats the achievement of health and health equity as preconditioned on addressing the wider social, economic and political inequalities that fuel it (Birn & Nervi, 2019; Giovanella et al., 2019; Sanders et al., 2019). It is to this discussion we hope to contribute by broadening the equity lens as it may apply to health system strengthening and providing some new avenues of linking equity to global health practice.

That contribution has two main thrusts. First, we provide a critical reading of key policy documents and secondary literature to trace the conceptual and normative development of health equity as a guiding principle for the WHO, while also contextualising equity’s shifting conceptual freight within the organisation’s changing policy landscape. Through this review, we hope to illustrate how equity has been taken up by the WHO not only as a central policy principle but also an operational measure and performance criterion. It is those efforts to *do* global health *equitably* – to translate the concept into global health practice – which, we argue, has reshaped and ultimately narrowed the way that the normative dimensions of equity – as a collective aspiration – is imagined.

Second, we seek to highlight the limitations in the current conceptualisation of equity in the framework of HSS at the WHO. For the past 20 years, the theme of health systems and HSS has played a central role the Organization’s efforts provide leadership in the field of global health. However, we argue that the way equity concerns have been operationalised as part of these efforts – as pertaining to the financing and distribution of healthcare or outcomes of discreet health interventions – risks curtailing the equity’s conceptual scope. Looking ahead, we point to new empirical avenues of equity research that may pave the way for a more ambitious HSS agenda that explicitly addresses health equity as part of a wider struggle towards social justice.

## Equity as a foundation to global health

In 1948, the World Health Organisation (WHO) was founded based on a constitutional commitment to ‘the highest attainable standard of health’ as a fundamental right of every human being (WHO, 1948), a commitment that was prominently affirmed in the demand for ‘health for all’ of the 1978 Alma Ata Declaration. Over the last 40 years, the Alma Ata Declaration and its ambitious equity agenda have provided a moral compass for the WHO. At the same time, how this quest for equity has been interpreted and made actionable has shifted considerably over this period. In what follows we describe that shift over three periods: Alma Ata as a major milestone in the bourgeoning field of global health that elevated health equity to a collective social goal; a subsequent period of growing international attention to health sector reforms that was accompanied by an increasing concern with allocative efficiency over equity; and developments since the millennium, where a notion of strong health systems as means to achieve health equity has gained increasing salience at WHO, in parallel to but largely de-coupled from, a revived attentiveness to the social, political and economic drivers of ill-health.

### Alma Ata and primary health care

In the 1978, Alma Ata Declaration asserted ‘health for all’ as a universal human right and declared the alleviation of health inequities a ‘main social target of governments, international organisations and the whole world community’ (WHO, 1978, §5). The Declaration pronounced the universal availability of essential health care as a bedrock of all health systems. But its radical force stemmed from the way it embedded demands for the better provision of healthcare within a much broader set of proposals for policy change: Primary Health Care (PHC) was to be at the heart of health systems ‘reoriented’ towards health promotion and prevention through community empowerment and intersectoral policies to tackle the socioeconomic and environmental problems underlying ill-health and health disparities (Mahler, 1981/2016). Achieving ‘health for all’, the Declaration asserted, had to be part of a much wider social justice agenda to upend the prevailing social and economic inequities and support countries’ and communities’ ‘development in the spirit of self-reliance and self-determination’ (WHO, 1978, §6).

The WHO itself described the Alma Ata Declaration as ‘synonymous with  … the quest for equity in health’ (WHO, 1988, p.3). Indeed, it provided the basis for WHO’s first explicitly ‘global’ strategy aimed at putting health at the centre of international development efforts: driven by then-WHO Director-General Halfdan Mahler, ‘Health for All by the Year 2000’ was billed as going beyond ‘the solution of purely medical problems, such as a lack of doctors, hospital beds, drugs and vaccines’ (Mahler, 1981/2016; as cited in Brown et al., 2016, p. 36). Instead, Mahler sought to establish WHO as the vanguard of an approach that treated health as much more than a medical matter and that explicitly acknowledged worldwide differences in health status as a reflection and catalyst of much wider systemic inequalities. Accordingly, health and health equity were not primarily treated as distinct, measurable target; rather, they were seen as a collective responsibility and orienting vision for national and international political commitment, intersectoral policies and societal transformation. As the WHO insisted at the time: ‘Health for all is therefore not a single, finite target; it is a process leading to the progressive improvement in the health of people‘ (WHO, 1981, p. 31).

The WHO would hold on to, and regularly re-affirm, its commitment to ‘Health for All’ over the next 40 years. And yet, fierce debates over this strategy, and over the orientation of the budding global health field more broadly, ensued almost immediately.

### The 1980s/90s: Equity vs allocative efficiency

As is well documented, many international organisations, governments and individuals dismissed ‘Health for All’ as too vague, idealistic and unattainable. Alternative strategies, such as Selective Primary Health Care, gained traction for proposing to focus on technical, targeted and result-oriented healthcare interventions whose success could be more easily evaluated (Birn, 2009; Cueto, 2004). And yet, these strategies also effectively severed international efforts to improve health and health equity from the social and political dimensions of PHC and the Alma Ata Declaration.

Under ongoing leadership from Mahler, the WHO positioned itself in these debates by at once defending its expansive social justice agenda and seeking to validate its continued support for HFA by pointing to areas of measurable success. Examples of this balancing act can be, for example, found in the report *From Alma Ata to the Year 2000* (WHO, 1988) published as part of a range of stock-taking activities at the 10-year anniversary of Alma Ata. On the one hand, the report proposed to use the coverage with PHC as a measure of equity. On the other hand, it reiterated that PHC should not be reduced to ‘only’ providing medical care and health services (ibid., p.15) and that ‘health for all’ went beyond quantifiable, technical goals. Rather, achieving health for all and health equity required a new ‘social imagination’ (ibid., p.35), a vision for health and healthcare as inseparable from broader social, economic and political development. But *From Alma Ata to the Year 2000* also marked the departure of Halfdan Mahler from WHO and, as such, an end to a period in which the organisation played what others have described as a highly politized role (Cueto, 2004), where health and health equity where explicitly pursued as part of a broader social justice agenda.

Indeed, the 10-year tenure of Mahler’s successor Hiroshi Nakajima was overshadowed by both internal controversy and a struggle for legitimacy as cuts to WHO’s budget were paralleled by the growing importance played by other organisations in the health field, especially the World Bank (Mahler 1981/2016). Published on the back of the Bank’s championing of structural adjustment and neoliberal macroeconomic reforms, its 1993 World Development Report *Investing in Health* (World Bank, 1993) spotlighted the importance of health for social and economic development. But the report was also coherent with the World Bank’s prevailing concern for broad policy reform aimed at improved efficiency and fiscal balance. *Investing in Health* heavily referenced the persistence of health inequities as a central challenge to be tackled by national and international efforts. However, it also framed the problem as primarily a failure of health systems and policies in developing countries: equity was described as one of a competing number of objectives for health sector reforms – with others including improved health outcomes and cost control (ibid., p.71) – which should be pursued by focusing government spending on a ‘minimum package’ of cost-effective clinical and public health interventions targeted at priority health problems.

Proponents hailed *Investing in Health* a landmark effort and its central tools, the population health measure Disability-adjusted Life Years (DALYs) and cost-effectiveness analyses, as innovative instruments for determining global health problems and their remedies. But for critics these tools lastingly re-configured what counts as valid targets and permissible solutions in global health discourses (Adams, 2016). Indeed, with regard to equity, the growing popularity of the DALY methodology is said to have fixed international attention on overall ‘global’ disease burdens at the expense of investigations into which conditions – and why – affect, for example, the global poor (Anand & Hanson, 1998; Gwatkin et al., 1999). More broadly, *Investing in Health* was an important catalyst for bourgeoning debates on health sector reforms in the 1990s in which the achievement of health equity was over-shadowed, if not all but abandoned, for the goal of achieving allocation efficiency within the healthcare sector (Blas & Hearst, 2002).

The WHO played an ambiguous role in these developments. While officially a collaborating partner on *Investing in Health*, other dedicated WHO initiatives, such as the initiative on Equity in Health and Health Care (WHO, 1996) and the Intersectoral Action for Health (IAH) project (WHO, 1997), tried to keep equity on the agenda and continued to emphasise that the provision of healthcare alone would be insufficient for the achievement of health equity. At the same time, however, the WHO struggled to retain funding and authority in a global health field where debates over the best organisation of health services increasingly took centre stage and where, in the process, ‘social imaginations’ of equity as a social and political goal seemed to become increasingly relegated.

### The Brundtland years and beyond: Health systems as vehicles towards measurable outcomes

In the late 1990s, Gro-Harlem Brundtland was elected WHO Director-General on a platform of proposals for wide-ranging reforms, including to the role played by the organisation on the international stage. In her first speech to the World Health Assembly, Brundtland outlined a broad strategic vision for the organisation’s strategy that seems aptly summarised by her use of a double entendre: ‘reaching goals based on values’ (Brundtland, 1998, p. 5). Indeed, on the one hand, her speech emphasised the importance of re-committing the WHO to the goal of ‘Health for All’ guided by a moral ‘imperative of equity and social justice’ (ibid., p.1). On the other hand, Brundtland also made clear that WHO’s focus should be on combating disease and ill-health by helping countries to ‘build sustainable health systems that can help reach equity targets and render quality services to all’ (ibid., p.3). In other words, the WHO should concentrate on pursuing health improvements as measurable goals to be achieved by targeted interventions to strengthen health systems.

This vision was elaborated further in subsequent reports published by the WHO. In her foreword to the first *World Health Report* produced under her directorship, for example, Brundlandt pointed to the lack of educational opportunities for girls, the environment and poverty as examples of the ‘[m]any determinants of better health lie outside the health system altogether’ (WHO, 1999, p.xiv). But, in a prognostication of what would become the central message of the Commission on Macroeconomics and Health that she set up a few months later, she also made clear that the priority of the WHO, and of the international health community more broadly, should be on investing in health systems and care as ‘one major avenue towards poverty alleviation’ (ibid., p.viii). In many ways, she thus turned one of the central components of the Alma Ata Declaration on its head: whereas the Declaration had advocated for the need to tackle poverty and other social, political and economic injustices as root causes that impeded improvements to health and health equity, Brundtland sought to shift attention to ill-health as ‘one of the root causes of poverty’ and on ‘health interventions that will help lead populations out of poverty’ (ibid., p.xi).

Indeed, the reform of health systems and services to optimise performance would become a major strategic focus of the WHO under Brundtland’s leadership. As part of this, as we will further argue below, the conceptual reach of equity was significantly curtailed. Indeed, the 1999 *World Health Report* offered a preview of this: although the terms ‘equity’ and ‘equitable’ feature about a dozen times, this is always in reference to the equitable distribution of or access to health services – rather than, for example, health status. Instead, the report repeatedly notes the alleviation of ‘excess disease burden’ among the world’s poor (ibid., p.xi) as a priority goal, to be achieved not by taking action outside the health sector, but by targeting public spending towards the equitable provision of specific, cost-effective health services for priority diseases (WHO, 1999).

These early speeches and reports laid the ideological grounds for what has become an increasingly prominent ‘health systems strengthening’ (HSS) agenda at WHO and beyond – an agenda whose potential we wish to argue remains somewhat constrained by the way that health equity considerations, if considered in depth at all, are typically reduced to the outcomes of healthcare services. It is important to note that we are, of course, not the first to identify these constraints; indeed, arguments for an intersectoral approach to achieving health equity have continued to be put forward, including by the WHO itself under Brundtland’s successors.

In what could have heralded a major re-centering of a more expansive WHO approach to health equity, the 2008 report by its Commission on Social Determinants of Health (CSDH, 2008) successfully mainstream the idea that people’s health should be seen as inseparable from their socio-economic position. Backed up by new empirical evidence on the impact of people’s living and working conditions on their health, the CSDH sought to re-focus international attention onto the need to address what it described as the root causes of ill-health and health inequities. In a nod to WHO’s strategic focus of previous years, the CSDH asserted the importance of strengthening health services and systems; however, it also insisted that these were but one determinant of health and that their improvement would have to take place within wider contexts of social, economic and cultural reforms. In the words of the CSDH report, to improve health and achieve health equity, the primary target had to be the ‘toxic combination of poor social policies and programmes, unfair economic arrangements, and bad politics’ (ibid., p.1).

The CSDH report’s outsized vision of health equity framed by a wider agenda for socio-economic justice drew comparisons to the Alma Ata Declaration and was celebrated by many commentators as a key step towards a more expansive conception of health equity (Venkatapuram & Marmot, 2009) and the revival of a momentum towards a wider social and political reform agenda (Baum, 2007; Birn, 2009). Indeed, by coupling normative concerns for health equity with a strong empirical evidence base for the social causes of ill-health, the CSDH report boosted monitoring of and advocacy for social determinants and health equity, as well as empirical and conceptual efforts to understand how health and health inequities are shaped by social inequalities as well as social policies and programmes, including at WHO (e.g. Hosseinpoor et al., 2015; WHO, 2015). And yet, we would argue that the parallel increase in attention and resources devoted to strengthening poorer countries’ health systems has taken place largely in isolation from these developments.

## Health systems strengthening and equity concerns

Work conducted at the WHO during Gro Harlem Brundtland’s tenure played an important role in turning ‘Health Systems Strengthening’ (HSS) into a key global health policy and research priority, amidst a growing rhetoric of poorer countries’ fragile health systems as critical ‘bottlenecks’ in the pursuit of international health targets and aid efficiency (Hafner & Shiffman, 2012).

One of the key catalysts was WHO’s *World Health Report 2000* (WHR 2000), which sought to set out new standards for assessing and improving health systems. Aligned with the broader strategic direction of the WHO under Brundtland, the *WHR 2000* emphasised the need to ‘clarify and quantify’ (ibid., p.xii) health system goals. Accordingly, it introduced a conceptual framework for health systems – defined as comprising ‘all the organizations, institutions and resources that are devoted to producing health actions’ (WHO, 2000, p. 5) – that identified health (as expressed though a derivative of the DALY measure), responsiveness and fairness in financial contribution as three universal and measurable health system goals. Further, it also offered strategies to improve performance against these goals that were targeted at four universal health system functions: service provision, resource generation, financing and stewardship. Importantly, the *WHR 2000* proposed that all three health systems goals should be captured not only in terms of the overall level but also in terms of their distribution, thus explicitly establishing equity as a quantifiable goal of health systems. And yet, the way it conceptualised and sought to measure equity was reflective of a rather narrow, overly technical and largely de-politicised equity agenda that had arguably taken hold at WHO.

Among equity experts, one of the biggest failings of the WHR 2000 concerned its proposal to measure health equity by estimating health differences between individuals rather than social groups, which they argued ‘effectively removes equity from the agenda for public health monitoring and policy’ (Braveman et al., 2001, p. 678). More critical for our argument, however, is that the WHR 2000 also conceptualised health equity in an exceedingly narrow way that continues to reverberate in the HSS agenda today. The report argued that

[t]he differing degrees of efficiency with which health systems organize and finance themselves, and react to the needs of their populations explain much of the widening gap in death rates between the rich and poor, in countries and between countries, around the world. (ibid., p.xii)

In designating the lack of efficiency in the delivery and financing of health services as the main cause of health inequalities, the WHR 2000 not only conflated allocative efficiency and equity. But it also re-emphasised health equity as primarily a technical issue to do with healthcare provision.

That limited view of health equity continues to haunt WHO’s health systems agenda, despite subsequent efforts by the CSDH and others to re-connect the strive to health equity to a more expansive social justice agenda. One key example is WHO’s, 2007 ‘Building Blocks’ framework (WHO, 2007), which echoes the claim of failing health systems as ‘at the centre’ of persisting ‘deep inequities in health status’ (ibid., p. 1). Even as the need for attention to wider ‘health determinants’ such as education and housing, is noted, these determinants are also deemed to be outside of – and separate from – the health sector. Instead, the building blocks framework reiterates the goal of more ‘equitable and sustained improvements across health services and health outcomes’ (ibid., p. 4) as primarily a function of equitable coverage of and access to healthcare services and products and for ‘fair’ health financing systems. In other words, at the same time that the strive for health equity has been upheld as a central goal of WHO’s HSS efforts, HSS debates have repeatedly reified health equity as a matter of healthcare delivery. This is not to say that there have not also been efforts to propose alternative health system frameworks that promote more nuanced understandings of the interactions between health systems and social determinants of health (Russell et al., 2013). And yet, the WHR 2000 and WHO’s ‘Building Blocks’ framework have shaped the health systems agenda (McKee, 2010) and continue to inform more recent taxonomies to define and classify HSS efforts (e.g. Kruk et al., 2018; Lavis et al., 2015).

Nowhere does this legacy become more apparent than with the most recent prioritisation of Universal Health Coverage (UHC), which both builds on the HSS agenda and arguably further exposes how the progressively narrowed focus on access to and outcomes of health services risk drowning out debates about a more expansive social justice agenda for the global health field. First adopted by the World Health Assembly in 2005, UHC aims at achieving both, *equity in access* to healthcare services and *equity in financing* (WHO, 2010). Inclusion in the *Sustainable Development Goals* has further solidified UHC as the global health field’s latest primary objective, with other goals – such as health system strengthening but also, as previously mentioned, PHC – often framed as means to an end (e.g. Kieny et al., 2017).

And yet, the blanket assumption that UHC programmes will lead to more equity is highly contested. First, as others have pointed out, truly universal healthcare access remains an elusive goal: even in countries purported to already have ‘universal’ healthcare systems, specific services (such as expensive treatments for cancer) tend to be excluded and certain groups (such as refugees) systematically disadvantaged (Birn & Nervi, 2019). And since UHC is typically conceived in terms of coverage with a circumscribed range of services, many healthcare services are likely to remain inaccessible for poorer population (Giovanella et al., 2019). Second, improving overall coverage of health services may initially benefit better off groups disproportionally and thus, at least initially, exacerbate existing health inequities (Gwatkin & Ergo, 2011; Victora et al., 2000). Third, existing evidence at country level suggests that, even after achieving UHC, disparities in health often remain largely unchanged, precisely because social determinants are unlikely to be addressed through an agenda confined to increasing coverage of basic health services (Arroyave et al., 2013). For many critics then, the push for UHC looks less like a rallying cry for transformative action and more like the latest instantiation of the ongoing biomedicalization of health and health systems (Birn & Nervi, 2019).

What these critical questions about UHC underscore, yet again, are the challenges of rendering practicable the ambitious goal achieving social justice, particularly when there is a lack of consensus on precisely what should be the moral object of concern – which inequities, in other words, can be acted upon and should be a matter for global health policy and practice. As a concept, equity may have broadly accommodated the various strategic and programmatic shifts at WHO since the Alma Ata Declaration, which themselves have reflected different leadership but also the wider political context in which the organisation has operated. And yet, what we have also sought to show is that at the same time that efforts have bourgeoned to define equity as a measurable goal of programmes and interventions – arguably in line with what other observers have described as a more general emphasis on quantification and metrics at WHO and the wider global health field (Adams, 2016) – this has come with a narrowing focus on the equitable access to or outcome from healthcare services at the expense of an emphasis on the wider social, economic and political forces that drive ill-health and shape the care-giving context.

## New empirical avenues

When it comes to actual policies and practices, ‘equity impacts’ continue to be rarely explicitly evaluated or reported (Rodney & Hill, 2014). This lack of empirical scrutiny also extends to the equity impacts of health systems strengthening interventions (HSSIs) (Ciapponi et al., 2017; Herrera et al., 2017). But while we agree that the strive for health equity needs to move beyond ‘hollow rhetoric’ (Shamasunder et al., 2020), we would equally insist that efforts to render equity a measurable and implement-able programmatic goal cannot come at the expense of narrowing its analytical purchase and political scope. This section discusses this tension at the example of a recently proposed ‘equity lens’, before providing a glimpse of the exciting opportunities that inhere in a broadened equity lens to guide HSS policies and practices.

In a key effort to routinise equity impact evaluations, the WHO recently joined a number of organisations, including the Cochrane Health Equity Group, in endorsing the PROGRESS framework as an ‘equity lens’ that should be routinely applied to the design and reporting of health interventions. Drawing on Margaret Whitehead’s definition of equity as ‘avoidable, unjust and unfair’ health differences, PROGRESS prompts researchers to consider variations in health outcomes among different population subgroups identified by a set of seven stratifiers: **P**lace of residence, **R**ace/ethnicity/culture/language, **O**ccupation, **G**ender, **R**eligion, **E**ducation, **S**ocio-economic status, and **S**ocial capital (O’Neill et al., 2014). Frameworks such as PROGRESS may indeed serve as useful ‘memory aids’ to help intervention planners target health interventions at vulnerable population groups or prompt evaluators and systematic reviewers to pay attention to the fact that health interventions may not benefit everyone equally. And yet, we would argue that these frameworks hardly suffice as an ‘equity lens’ for health and health systems interventions.

For once, such standardised frameworks cannot supplant the challenging task of determining if differential distributions of health outcomes in particular contexts are indeed unfair or unavoidable. Furthermore, any uncritical adoption of such standardised frameworks also risks overlooking the constructed nature of social categories such as gender, race and social status; the fact that these categories operate differently across specific contexts; and the performative nature of social categories as they necessarily make certain disadvantages visible while obscuring others (Adams et al., 2019). For example, PROGRESS ignores a much wider range of structural factors – including racism, colonialism, sexism, ageism, ableism, classism, homophobia and transphobia – that have been shown to underlie health inequalities (Bailey et al., 2017; Krieger, 2020). Whereas PROGRESS may thus enable subgroup analysis for epidemiological purposes, or even the identification of a small set of common individual-level risk factors for disadvantage, it has little to say about how to identify and address the systems of privilege and oppression that undergird health disadvantages, about how forms of inequity intersect and become embodied, and about the types of multi-sectoral actions that may be needed to address resulting health inequities. Indeed, that proponents of PROGRESS blithely declare the principle aim to be to ensure that ‘the disadvantaged do not miss out on health benefits of interventions’ (O’Neill et al., 2014, p. 62) highlights the inadequacy of such routine ‘equity lenses’ as a panacea to addressing health inequalities.

Of course, not all health interventions may be able to tackle root causes of health inequities. And yet, when promoted as ‘equity lenses’, frameworks such as PROGRESS nonetheless risk further reducing equity considerations to concerns about equal access to, and outcomes from healthcare services. For once, they arguably risk reifying health equity as a post-hoc distributional issue whereby the focus is on detecting differential intervention effects across PROGRESS subgroups which, while important, is different from explicitly embedding equity considerations in the design of interventions. Conversely, biomedical technologies and public health initiatives may prove to be ‘equity focused’ under the PROGRESS framework, e.g. by being targeted at a population identified as disadvantaged, and yet embed forms of oppression and reproduce or even ‘innovate inequities’ (Benjamin, 2016). To expand on an example provided by O’Neill et al. (2014) themselves: ‘culturally appropriate’ health education may be effective in prompting lifestyle changes among ethnic groups deemed at increased risk for Type 2 diabetes; yet it may also risk overemphasising both cultural difference and lifestyle choices at the expense of structural issues, such as poverty, the built environment, environmental pollution, accessibility of nutritious food, and structural barriers to accessing care (Carpenter-Song et al., 2007; Hill-Briggs et al., 2020). In regard to health systems strengthening (HSS) interventions, an additional caveat is that establishing the impact and ‘system-wide’ effects of such interventions has shown to be challenging (Adam et al., 2012; Witter et al., 2019). As a result, the PROGRESS framework may struggle to capture even short-term equity gains directly linked to anticipated health-related intervention outcomes, let alone wider, long-term and even unintended*,* equity-relevant consequences of HSS interventions.

### Resituating equity

How the health systems agenda might be transformed to (re-)situate the strive for health equity within a broader social justice agenda is, needless to say, a project of profound complexity. However, we wish to point to two potential avenues of interrogation. The first is simply to broaden the equity lens to examine the experience and impacts of the health work force. Deemed by the WHO to be a core ‘building block’ of all health systems, health workers have become a primary target of HSS efforts and ‘task shifting’ a classic example of a widely-used HSS intervention (WHO, 2008). And yet, in contrast to a solid evidence base on the improvement of access to selected health services as an outcome of the redistribution of tasks among health worker teams, there is limited evidence that shows that task shifting improves inequities, or indeed little agreement as to how such inequities should be defined and assessed (Orkin et al., 2018).

With a growing body of evidence suggesting that task shifting may exacerbate health workers’ perceptions of being overburdened, unprepared and underpaid (Mijovic et al., 2016), one potential concern is that task shifting compromises health workers’ ability to deliver high-quality care, and may thus have negative distributional impacts on access to health care services and health outcomes. But, more so, we would argue that there is a lack of sustained debate on how such interventions may have negative equity-relevant impacts on health workers and health systems more broadly. For once, task shifting raises important questions about how efforts to strengthen health systems compete with other normative goals, such as ensuring and safeguarding adequate working conditions for health workers. Indeed, that inequities may result from ‘[e]xposure to unhealthy, stressful living and working conditions’ was a central component of Margaret Whitehead’s equity definition (Whitehead, 1991, p. 219), and the CSDH’s recommendations. Considering health workers’ wellbeing as part of the outcomes that HSS initiatives should aim to promote, alongside equally important goals such as narrowing inequities in health care and outcomes for patients, may thus be a first but necessary step in broadening the equity lens for HSS. Health workers tend to live in and are part of the communities that they serve. Building on this insight, which was central to Alma Ata, will require moving beyond conceiving of health workers as mere ‘building blocks’ and for international research and treatment efforts to stop instrumentalise them as vehicles for the achievement of short-term desired project outcomes. What is needed instead are long-term efforts to foster working and living environments that allow health workers to *care*.

This leads to a second avenue to broaden the equity lens for HSS, which derives from the pressing imperative to address the myriad ways in which the global health field itself builds on and reflects social injustices. Much work remains to be done to expose how histories of colonialism and their legacies enduringly undergird imbalances in resources and power and forms of oppression that not only underlie health inequities but also continue to pattern the global health field itself. Global North experts continue to dominate leadership positions at major global health agencies, hold sway over international research collaborations, and shape programme and research priorities (Boum et al., 2018; Crane, 2013; Okeke, 2018). In fact, international commitments to support the strengthening of health systems arose, at least in part, to critiques of donor-driven, short-term, disease-focused interventions that have circumvented, if not further contributed to the fragmentation of, poorer countries’ health systems (Hafner & Shiffman, 2012). And yet, not only does the health systems agenda lack behind its promises (Shamasunder et al., 2020). But the way that equity is progressively rendered as a technical goal linked to the provision of health services, as we have sought to argue in this paper, also threatens to decouple these commitments from a broader conversation over who gets to shape the global health agenda. The growing tide of calls for the ‘decolonialisation’ of global health injects a new urgency into these debates: as has been argued, it is only by ‘changing who sits at the table and rebuilding parts of the table itself’ that the global health field can start to address the way that structural inequalities that are at the root of health inequalities are also ‘reproduced within the global health system itself’ (Büyüm et al., 2020, p. 3).

While these brief suggestions cannot do justice to the many debates that are already ongoing they nonetheless highlight the profound need and potential for a broadened equity lens in the context of ongoing debates on health systems and the need to strengthen them. It is students who are at the forefront of demanding a ‘more radical and political take on the “progressive” framework of global health equity’ (Koris et al., 2020). We would suggest that it is time for the field to take up the challenge.

## Conclusion

In this paper, we have argued for the importance of re-considering what health equity implies in the context of health systems strengthening. Tracing the conception of equity at key periods in WHO’s history, we cautioned against what we suggested to be an increasingly unidimensional conceptions of equity as being a problem of either unequal access to specific healthcare services, or the differential health impacts of specific health interventions. It is beyond dispute that ensuring that all people have access to the healthcare they need must be a crucial component of efforts to improve health equity. However, a HSS agenda that focuses predominantly on improving health service delivery falls short of considering the structural political, social and economic dimensions that drive and sustain ill health and health inequities worldwide.

In fact, that strengthening health systems and improving health services are necessary but not sufficient conditions for mitigating ill-health and health inequities are insights that the WHO has helped popularise, not least through Alma Ata, its championing of Health for All, and in setting up the Commission on the Social Determinants of Health. And yet, as the focus has shifted to health system strengthening and, more recently, to UHC, key insights from these initiatives seem to have been forgotten or relegated. This paper focused on three lines of enquiry that may help enriching conceptions of equity for HSS: first, we need to replace simplistic standardised frameworks to measure equity with broadened frameworks that identify intersecting forms of social disadvantage in particular contexts. Second, a first step in rethinking equity could involve addressing the equity questions that arise in relation to health workers in the context of HSS. Third, we concur with the need to re-focus attention onto the imbalances in resources and power and forms of oppression that undergird health inequities – and shape the global health field itself.

Encouraging these kind of debates and returning to a broad-based vision of health systems as catalysts for the social, economic and political wellbeing of communities, and as the basis for better and fairer global health, would help WHO to re-assert its moral leadership and justify the claim it stakes on the ‘organizational embodiment of the commitment to equity’.
